# Catastrophic Antiphospholipid Syndrome: A Life-Threatening Condition

**DOI:** 10.7759/cureus.64367

**Published:** 2024-07-11

**Authors:** Sonal Prasad, Jay Xiong, Edsel Embry, Loui Abdelghani

**Affiliations:** 1 Department of Internal Medicine, St. Joseph's Medical Center, Stockton, USA; 2 Department of Internal Medicine, St. Joseph’s Medical Center, Stockton, USA; 3 Department of Pulmonary and Critical Care Medicine, St. Joseph's Medical Center, Stockton, USA

**Keywords:** autoimmune thrombocytopenia, thrombotic micro-angiopathy, autoimmune disoders, antiphospholipid antibody syndrome (aps), catastrophic antiphospholipid syndrome (caps)

## Abstract

Antiphospholipid syndrome (APS) is characterized by thrombosis in any organ or tissue, accompanied by the presence of antiphospholipid antibodies. Although rare, APS can progress to catastrophic APS (CAPS), a life-threatening complication involving the development of multi-organ thromboses. The mortality rate is high. Treatment consists of triple therapy with anticoagulation, glucocorticoids, and therapeutic plasmapheresis or intravenous immunoglobulins. We present a case of a patient with CAPS, requiring a multidisciplinary team approach to help diagnose and treat this complex disease.

## Introduction

Antiphospholipid syndrome (APS) is an autoimmune disorder that can cause thrombosis in any part of the organ system. The most common areas are the lower extremities and cerebral arterial circulation. It is also often associated with recurrent pregnancy loss. The incidence of APS is about 2.1 in 100,000 individuals in the United States [1]. A rare sequela of APS is CAPS. It occurs in less than 1% of patients with APS and mortality rates are as high as 50%. Unlike APS, CAPS is characterized by multi-organ thrombosis within a short period, which is also known as a *thrombotic storm* [1-3]. This is usually due to an increase in inflammatory response. The most common trigger is infection followed by surgery, anticoagulation discontinuation, medication, obstetric complication, and finally neoplasm [3-4]. About 72% of patients with CAPS are women with a mean age of 37. Other autoimmune diseases such as systemic lupus erythematosus or lupus-like syndrome may be present as well [4]. Common symptoms are fever, thrombocytopenia, acute renal failure, neurological deficits, and disseminated intravascular coagulation [3]. A multidisciplinary team consisting of various specialties is often necessary due to the complexity and life-threatening nature of the disease [2]. The most difficult thing about CAPS is that several patients present with CAPS but do not have any previous diagnosis of APS. This makes it challenging to diagnose on presentation, causing delays in treatment. In a study of 280 patients from the *CAPS Registry*, it was shown that about half the patients had a prior diagnosis of APS and the other half were newly diagnosed [5]. In this study, we report a rare case of a patient who presented with CAPS. She did not have any prior diagnosis of APS. A multidisciplinary team of multiple specialists was required to promptly diagnose and treat the patient with steroids, anticoagulation, and plasmapheresis.

## Case presentation

**Figure 1:**
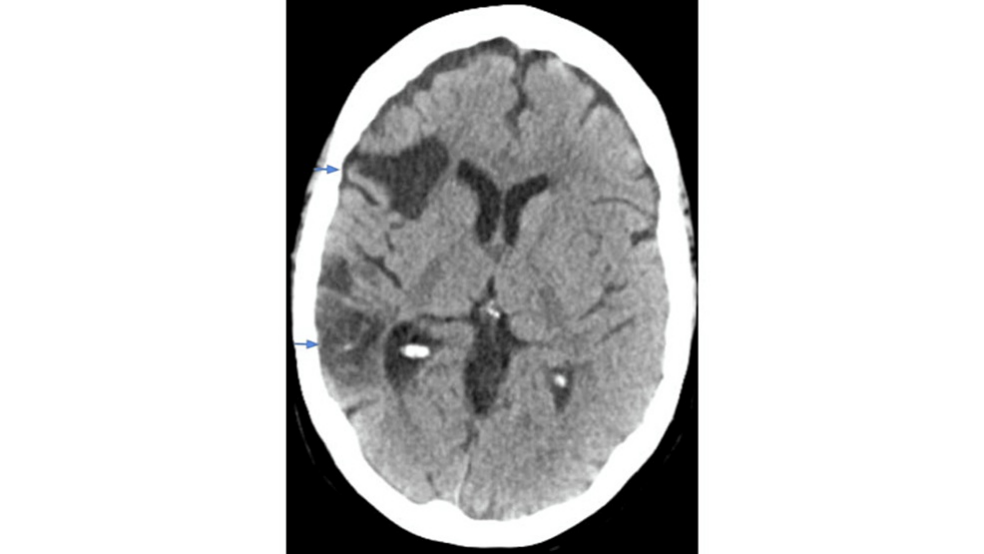
Large chronic right middle cerebral artery territory infarct (blue arrows).

A 61-year-old female with a medical history of coronary artery disease status post-coronary artery bypass graft, bioprosthetic mitral valve replacement, heart failure with preserved ejection fraction, hypertension, chronic obstructive pulmonary disease, obstructive sleep apnea, cerebral vascular accident with no residual deficits, and seronegative rheumatoid arthritis previously on Methotrexate, presented with altered mental status, difficulty ambulating, hearing loss, skin changes, and decreased oral intake. A history of present illness was obtained from the patient's son as she was confused. According to her son, the patient suddenly became confused and experienced difficulty walking and hearing, along with skin discoloration on her legs. She also had a productive cough with yellowish sputum and a poor appetite for a few days. She denied fever, chills, chest pain, nausea, vomiting, diarrhea, or dysuria. On presentation, vital signs were significant for a heart rate of 127 beats per minute, a respiratory rate of 41 breaths per minute, and the patient required 4 liters of oxygen via nasal cannula. Physical examination was notable for altered mentation, oriented to her name only, reduced breath sounds in bilateral lung fields, and purpura on her lower extremities. Initial labs showed a white blood cell count (WBC) of 3.1 thousand/uL, hemoglobin (Hgb) of 7.1 gm/dL, hematocrit (Hct) of 21.7%, platelets of 87 thousand/uL, blood urea nitrogen of 52.2 mg/dL, creatinine of 3.4 mg/dL, and an estimated glomerular filtration rate of 15 mL/minute. Urinalysis was significant for greater than 182 red blood cells and 3+ blood. Computed tomography (CT) of the head showed no acute intracranial hemorrhage or extra-axial fluid collection. It did reveal microvascular ischemic disease along with a large chronic infarct in the territory of the right middle cerebral artery (Figure 1).

The patient was admitted for sepsis secondary to pneumonia, acute kidney injury, and acute metabolic encephalopathy. She was given intravenous (IV) fluids and started on IV antibiotics. Her mental status improved the following day, and she provided more details such as having a history of multiple miscarriages, discovering new skin changes on her legs, and having difficulty hearing. Unfortunately, the patient was unable to specify the number of miscarriages and gestational weeks at which they occurred. Given the previous history of autoimmune disease, nephrology, hematology, and rheumatology were all eventually consulted.

The patient had a full workup done for her anemia and thrombocytopenia. Differentials included thrombotic thrombocytopenic purpura versus hemolytic uremic syndrome versus autoimmune hemolytic anemia versus immune thrombocytopenia versus acute illness-related thrombocytopenia versus antiphospholipid syndrome. Labs revealed elevated reticulocyte count, elevated lactate dehydrogenase (LDH), and low haptoglobin levels. The iron panel showed anemia of chronic inflammation with elevated ferritin and low iron saturation. The fibrinogen and coagulation panel did not show any evidence of disseminated intravascular coagulation. The Direct Coombs test, paroxysmal nocturnal hemoglobinuria panel, and ADAMTS13 screen were all negative. Peripheral blood smear showed no schistocytes ruling out microangiopathic hemolytic anemia processes. Further workup revealed low complement 3 and complement 4 levels and negative antineutrophil cytoplasmic antibodies (ANCA), antinuclear antibodies, and double-stranded DNA antibodies, ruling out ANCA-associated vasculitis and systemic lupus erythematosus. The antiphospholipid antibody profile result was triple positive for beta-2 glycoprotein 1 antibodies, anti-cardiolipin antibodies, and lupus anticoagulants. The low complement levels, urinalysis with greater than 182 red blood cells and 3+ blood, and peripheral blood smear showing no schistocytes all pointed toward concern for microvascular thrombotic microangiopathy (TMA). Table 1 summarizes the patient’s laboratory results.

**Table 1:** Laboratory results summary. INR, international normalized ratio; PT, prothrombin time; PTT, partial thromboplastin time; PNH, paroxysmal nocturnal hemoglobinuria

Labs	Range	Reference ranges
Absolute reticulocyte count	166.2 thousand/uL	20-150 thousand/uL
Reticulocyte count	7.49%	0.5%-2.5%
Lactate dehydrogenase	410 units/L	125-243 units/L
Haptoglobin	43 mg/dL	63-273 mg/dL
Fibrinogen	453 mg/dL	200-393 mg/dL
INR	1.4	<1
PT	16.5 seconds	9.4-12.5 seconds
PTT	36.0 seconds	25.1-36.5 seconds
Direct Coombs test	Negative	Negative
PNH panel	Negative	Negative
ADAMTS13	0.58 international unit/mL	0.68-1.63 international unit/mL
Complement component 3	58.0 mg/dL	82-193 mg/dL
Complement component 4	5.6 mg/dL	14-53 mg/dL
Myeloperoxidase antibody	2 arbitrary units/mL	0-19 arbitrary units/mL
Serine protease 3 antibody	0 arbitrary units/mL	0-19 arbitrary units/mL
Antinuclear antibody	<1:80	<1:80
dsDNA Ab, IgG	12 international units/mL	0-24 international units/mL
Beta-2 glycoprotein 1 antibody IgG	>150	<20
Beta-2 glycoprotein 1 antibody IgM	>150	<20
Anti-cardiolipin IgG antibody	20	<14
Anti-cardiolipin IgM antibody	>150	<12
Lupus anticoagulant	Positive	Negative

Renal biopsy showed immune complex-mediated glomerulonephritis with membranoproliferative features as well as glomerular fibrin thrombi, consistent with focal thrombotic microangiopathy. Per nephrology and rheumatology, these findings were likely due to antiphospholipid syndrome with renal involvement, given the patient’s clinical history and laboratory findings.

In the setting of new-onset purpura, thrombocytopenia, anemia, hearing loss, confusion, ataxia, worsening acute renal failure with biopsy showing immune complex-mediated glomerulonephritis with membranoproliferative features and thrombotic microangiopathy, low complement levels, and triple-positive antiphospholipid antibodies (APLA) profile, microvascular TMA in the setting of CAPS became high on our differential. The patient was treated with pulse dose steroids of IV methylprednisolone 1,000 mg daily for the first three days followed by prednisone 60 mg daily, plasmapheresis, and IV unfractionated heparin drip with transition to warfarin. The patient responded well to the treatment.

## Discussion

APS is an autoimmune disorder that is characterized by thrombosis in any part of the tissue or organs in addition to having positive antiphospholipid antibodies [1,6]. To diagnose someone with *definite APS*, they need to satisfy at least one clinical criteria and one laboratory criteria. One of the clinical criteria consists of at least one episode of vascular thrombosis in any tissue or organ. The other clinical criterion relates to pregnancy morbidity. For example, if there is at least one unexplained death of a morphologically normal fetus at or beyond the 10th week of gestation, or at least one premature birth of a morphologically normal neonate before the 34th week of gestation due to eclampsia or other recognized features of placental insufficiency, or at least three unexplained consecutive spontaneous abortions before the 10th week of gestation [6]. These antiphospholipid antibodies can cause arterial or venous thrombosis or pregnancy loss. The laboratory criteria consist of having a lupus anticoagulant, anticardiolipin antibody, or anti-beta-2-glycoprotein-I antibody that is permanently present in the plasma [1,6]. The antibodies can be identified using an enzyme-linked immunosorbent assay (ELISA) or functional clotting assays [1]. Our patient has a history of multiple miscarriages, large chronic right middle cerebral artery territory infarct, bioprosthetic mitral valve replacement, and she presented with new onset purpura on her lower extremities, acute renal failure, thrombocytopenia, hearing loss, confusion, and ataxia. She was triple positive for APLA.

In order to diagnose someone with CAPS, they must satisfy four criteria: (1) involvement of at least three organ systems; (2) development of manifestations simultaneously or within a week; (3) histopathological confirmation of small-vessel occlusion in at least one organ; and (4) laboratory confirmation of the presence of APLA. If all four criteria are satisfied, then the patient is listed to have *definite CAPS*. If only three criteria are satisfied, then it is *probable CAPS* [1]. The classification of *probable CAPS* is intended to prompt physicians to remain vigilant and swiftly diagnose and treat the patient before the condition progresses to *definite CAPS* [6]. Our patient presented with involvement of vascular, cutaneous, hematological, neurological, and renal organ systems, all developing within a short span of time. In addition, laboratory results showed triple positivity for lupus anticoagulant, anticardiolipin antibody, and anti-beta-2-glycoprotein-I antibody, along with histopathological confirmation of TMA in the renal glomeruli as mentioned earlier. Based on the classification criteria, she needed the antibody test to be repeated at least 12 weeks later to be classified as *definite CAPS*. At the time of reporting, she was *probable CAPS*, and if the antibody was negative 12 weeks later, she would have been classified as *probable CAPS*.

Treatment of CAPS usually requires a multidisciplinary approach with different specialists due to the complexity and life-threatening nature of the disease. Our team involved hematology, rheumatology, nephrology, and critical care. There is no single guideline in the management of CAPS as it is very patient specific. Decisions are revised numerous times due to the constant changes in the patient’s clinical status and laboratory results. Management is usually with a combination of anticoagulation, glucocorticoids, and therapeutic plasmapheresis or IV immunoglobulins. This is sometimes referred to as triple therapy [6]. Rodríguez-Pintó et al. compared the effects of triple therapy versus drugs included in the triple therapy but in different combinations versus no treatment in patients with CAPS. The mortality rates were 28.6%, 41.1%, and 75%, respectively. Basically, triple therapy was positively associated with a higher survival rate than the other two groups [7]. We used triple therapy for the management of our patient: pulse dose steroids of IV methylprednisolone with tapering, plasmapheresis, and IV unfractionated heparin drip with transition to warfarin. In a study by Erkan et al., it was noted that 66% of the patients who survived CAPS remained symptom-free with anticoagulation during an average follow-up of about 67 months, whereas 26% developed further APS symptoms [8].

## Conclusions

Although CAPS is rare, patients with APS should be cautiously monitored for any signs and symptoms of simultaneous multi-organ thrombosis. High vigilance is needed for prompt diagnosis and management due to its significantly high mortality rates. Additionally, patients with multiple histories of unprovoked thrombosis or recurrent pregnancy losses should be screened for antiphospholipid syndrome. Our patient had a significant past medical history that could potentially have indicated APS, but she never underwent a workup. For example, she had a history of multiple miscarriages and a large chronic right middle cerebral artery territory infarct. Early diagnosis and treatment of CAPS can improve overall outcomes. All patients should be treated with the triple therapy combination to help increase their survival rates.
